# Editorial: Hepatitis B virus and host interactions in liver diseases

**DOI:** 10.3389/fcell.2023.1204280

**Published:** 2023-05-04

**Authors:** Yuen Gao, Xiao-Jie Lu, Yaoping Shi

**Affiliations:** ^1^ Department of Physiology, Michigan State University, East Lansing, MI, United States; ^2^ First Clinical Medical College, Nanjing Medical University, Nanjing, China; ^3^ Renji Hospital, School of Medicine, Shanghai Jiao Tong University, Shanghai, China

**Keywords:** hepatitis B virus, mitochondria, cell senescence, nucleos(t)ide analogs, interferons

## Introduction

Chronic hepatitis B virus (HBV) infections, leading to approximately 650,000 fatalities annually, is a major global health burden. Understanding the interaction between HBV and host liver cells will contribute to develop diagnosis and treatment of liver fibrosis, cirrhosis, and hepatocellular carcinoma (HCC). Mitochondrial dysfunction is a common feature that results from viral infection, causing the accumulation of reactive oxygen species and the activation of cell senescence. In this Research Topic, innovative approaches are applied to explore the function of mitochondria and cell senescence in liver disease progression, and to monitor the effectiveness in antiviral treatment. Finally, five interesting original research articles are accepted in this Research Topic.

## Mitochondrial function and HBV infection

Mitochondrial function plays a critical role in cellular processes, but the altered mitochondrial function in HBV infection progress is not clear. Lin et al. present promising findings that serum Adenosine Triphosphate (ATP) could serve as a potential diagnostic biomarker for chronic HBV infection and its related diseases. The research demonstrates that lower levels of serum ATP are significantly associated with HBeAg-positive chronic HBV patients, while increased levels are observed during the progression of chronic HBV to liver cirrhosis and HCC. The study further provides preliminary insights into the underlying mechanism behind the decline in serum ATP, which points towards impaired mitochondria in chronic HBV patients. These findings have the potential to improve the clinical diagnosis and management of chronic HBV infection, which can be challenging due to the need for liver biopsy. Overall, this study highlights the importance of exploring alternative mitochondrial biomarkers for improving disease diagnosis and management.

## Cell senescence and HBV infection

Senescent cells display a distinct phenotype, including changes in morphology, gene expression, and secretion of pro-inflammatory factors known as the senescence-associated secretory phenotype. The association between cellular senescence and HBV infection, as well as the potential nomogram (calculating the value of one variable based on the values of other related variables), remains undetermined. Yu et al. provide important insights into the role of cellular senescence in shaping the immune microenvironment and affecting the prognosis of HBV-related HCC. The identification of two distinct cellular senescence subtypes and the establishment of a cell senescence (CS) score signature based on five genes is a significant contribution to our understanding of the molecular mechanisms underlying HBV-related HCC. The findings of this study have the potential to inform the development of personalized immunotherapy for HBV-related HCC patients, and the CS dynamic nomogram could be a valuable tool for predicting patient prognosis. Together, this study highlights the importance of considering cellular senescence in the development of new therapeutic strategies for HBV-related HCC.

## Antiviral treatment and beyond

Antiviral treatment for hepatitis B virus (HBV) infection plays a crucial role in managing and preventing the progression of HBV-related liver diseases. The primary goal of antiviral therapy is to suppress HBV replication, reduce liver inflammation, and prevent or reverse liver damage. Currently, nucleos(t)ide analogs and interferons are the two main classes of antiviral drugs used in the treatment of chronic HBV infection.

Yang et al. provide important evidence for the effectiveness of interferon add-on therapy in achieving clinical cure for interferon-experienced patients with low HBsAg. The findings suggest that patients who have poor responses to their first interferon therapy and are subsequently switched to nucleos(t)ide analog therapy may still benefit from interferon add-on therapy if their HBsAg levels drop to a low level. The study reports that the week 48 HBsAg clearance and seroconversion rates were similar between interferon-experienced and interferon-naive patients, indicating a satisfactory clinical cure of the interferon add-on therapy for interferon-experienced patients.

Yuan et al. highlights the importance of various cytokines, including MMP-1, CXCL9, CXCL10, and TNF-R1, in the loss of HBsAg among chronic HBV patients. The findings of this research can be useful for the development of a nomogram that takes into account these factors to predict HBsAg loss. However, further research is needed to understand the immune processes involved in HBsAg loss. The positive relationship between interferon treatment and HBsAg loss suggests its potential as a treatment option for chronic HBV patients. The negative correlation between baseline HBsAg levels and HBsAg loss implies that early detection and treatment of chronic HBV may improve the chances of achieving functional cure.

Shi et al. present a useful nomogram for predicting the incidence of HCC in patients with hepatitis-B related cirrhosis who are receiving antiviral treatment. The nomogram, based on three independent risk factors, showed good performance in both the derivation and validation cohorts. The study emphasizes the importance of surveillance for high-risk patients with a score of over 10 points. The development of such a nomogram can help in the early detection of HCC, which can significantly improve patient outcomes. Overall, this study highlights the importance of risk prediction models in clinical practice and can aid in the development of personalized management strategies for patients with HBV related cirrhosis.

In this Research Topic, we focus on understanding the molecular mechanisms allowing HBV to hijack the host cell and destroy liver tissue. Novel therapeutic approaches are required to target to different aspects of the HBV life cycle, eliminate covalently closed circular DNA, and provide a functional or complete cure for HBV infection. Ongoing research focuses on developing new antiviral agents, host-targeting agents, and immunotherapeutic strategies to achieve these goals.

